# Isolated Esophageal Injury Following Blunt Thoracic Trauma: A Rarity

**DOI:** 10.4021/gr2009.10.1317

**Published:** 2009-09-20

**Authors:** Satish Dalal, Nityasha Dalal, Pawan Goyal

**Affiliations:** a Department of General Surgery, Pt.B.D.Sharma Postgraduate Institute of Medical Sciences (P.G.I.M.S.), Rohtak-124001, Haryana, India

**Keywords:** Blunt injury, Esophageal tear, Thoracotomy

## Abstract

Esophageal injury following blunt trauma to chest is an extremely rare event, with only a limited number of cases being reported in the world literature. We report a case of perforation of the lower thoracic esophagus following a crush injury to the chest in a 14 year old child. An appropriately placed chest drain and decompression gastrostomy resulted in complete resolution of the esophageal leak within four weeks. This case report demonstrates that a conservative approach to lower thoracic esophageal perforations can be carried out successfully without the added morbidity of thoracotomy and risks of direct repair.

****

## Case Report

**Figure 1:**
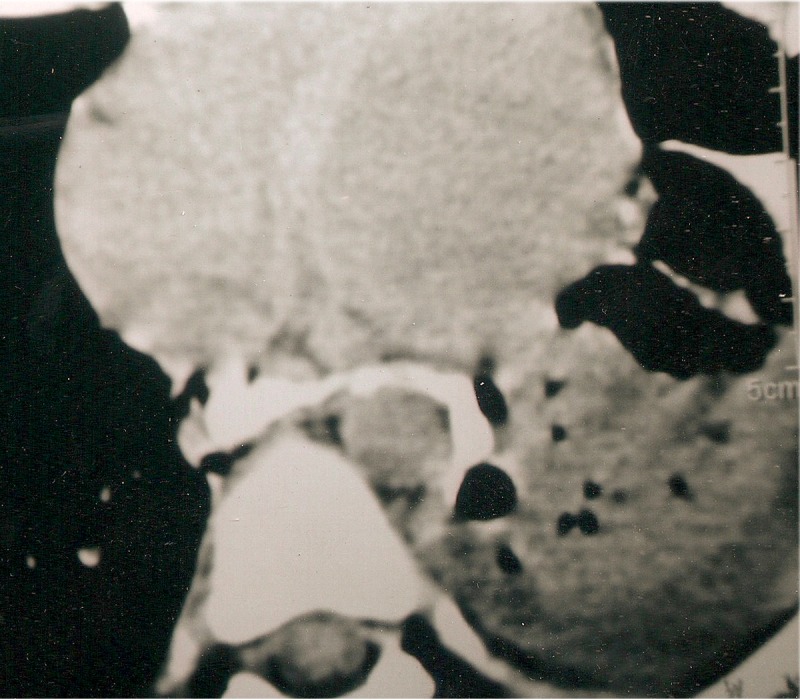
CECT thorax film showing leakage of contrast from lower esophagus into left pleural cavity.

A 14 years old, male child was admitted to our hospital two days after a road accident with clinical diagnosis of chest injury. Immediately after the accident, he got consultation from a private practitioner thinking it to be a minor injury. Subsequently he developed increased chest pain along with breathlessness. On admission, his pulse was 102/minute and respiratory rate was 24/minute. He had abrasions over left chest wall and sternum but no palpable fracture ribs. Auscultation of chest revealed decreased air entry on the base of left lung. An intercostal drain was put in left pleural cavity, which drained about 500 ml fluid containing saliva and milk. An esophageal tear was suspected and CECT chest was performed for confirmation, which showed mediastinal collection and contrast trickling from lower esophagus into the left pleural cavity (Fig. 1).

A decompression gastrostomy was done with 28 Fr abdominal drain catheter which was passed into the stomach and was negotiated into the esophageal lumen through the esophagogastric junction, primarily for drainage of esophagus at the perforation site. An extra hole was fashioned in the tube to drain the stomach. Upper G.I. endoscopy was done to exactly confirm the position of the gastrostomy tube at the level of perforation. Additional feeding jejunostomy was done. In the postoperative period, patient was given injection Cefotaxime and Amikacin for one week and then dispersible Cefexime tablets through jejunostomy. After 48 hours, jejunostomy feeds were started which were gradually increased to his normal daily calorie and protein requirements. The patient was nursed in a sitting position to prevent the regurgitation of gastric contents. He made an uneventful recovery and the intercostal drain and the gastrostomy tubes were removed after three weeks. Contrast esophagogram done after four weeks revealed a nearly normal esophagus. Normal oral intake was then introduced without any problems and patient was discharged home.

## Discussion

The most common cause of esophageal trauma is that which follows an endoluminal procedure, such as endoscopy, dilatation or transesophageal ultrasound, etc. [1]. Thoracic esophageal rupture secondary to blunt trauma is an exceedingly rare injury. In a meta-analysis carried out in 1988, Beal et al identified 96 cases from 1900 to 1988 [1]. Of these they reviewed 63 cases and only five cases (7.9%) of them had lower thoracic perforations. The mechanism of injury was most often from rapid acceleration / deceleration injuries [1]. Rupture due to raised intraluminal pressure from abdominal compression in the presence of a closed glottis, is the other mechanism proposed by Stothert et al [2]. Review of published reports also revealed two cases of isolated esophageal perforation after minor trauma as a direct result of the Heimlich manoeuvre [3].

The clinical features are frequently delayed and a high index of suspicion is required to establish the diagnosis. The clinical features may be tachypnoea, retrosternal pain, surgical emphysema and pneumomediastinum on x-ray chest. In suspected cases, diagnosis can be confirmed either by CECT thorax or by contrast esophagogram with water soluble dyes. In our case the diagnosis was confirmed by CECT chest. Management options in esophageal perforations include primary repair with drainage, drainage alone, esophageal exclusion with drainage or resection with cervical esophagostomy [4]. The optimum strategy for managing lower thoracic esophageal perforations following blunt trauma is unclear because of rarity of such cases. Cordero et al in 1997 reported two such cases that were managed by primary repair; but persistent leak from the anastamosis in one of the case necessitated esophageal exclusion [5]. Endoluminal drainage via gastrostomy and extraluminal drainage via a well placed chest drain combined with enteral nutritional support have been shown to have similar success rates to thoracotomy and primary repair [6]. Same method of treatment was adopted in our case and it led to a successful outcome with avoidance of thoracotomy.

Through this case report, the author wants to emphasize the following points: (1) Since it is an extremely rare injury, a high index of suspicion is required for diagnosing lower esophageal perforation after blunt chest trauma; (2) Adequate drainage via gastrostomy and chest drains can be safe and effective in such patients without added morbidity of a thoracotomy.
